# Effect of health education on knowledge of home management of diarrhoea amongst caregivers of under-five children in Yenagoa, Nigeria

**DOI:** 10.11604/pamj.2023.46.46.40904

**Published:** 2023-09-29

**Authors:** Njideka Mesiobi-Anene, Joseph Ezeogu, Emmanuel Okechukwu Anene, Chika Onyinyechi Duru, Peterside Oliemen, Akinbami Felix Olukayode

**Affiliations:** 1 Department of Paediatrics and Child Health, Niger Delta University Teaching Hospital Okoloibiri, Bayelsa State, Nigeria,; 2 Department of Paediatrics, Federal University Teaching Hospital Owerri, Imo State, Nigeria,; 3 Department of Internal Medicine, Niger Delta University Teaching Hospital Okoloibiri, Bayelsa State, Nigeria

**Keywords:** Diarrhoea, under-fives, caregivers, home management

## Abstract

**Introduction:**

low knowledge level of diarrhoea treatment, and appropriate use of oral rehydration therapy by caregivers, has been attributed to delay in commencing home management of diarrhoea. This delay in commencing home treatment; has led to preventable loss of lives arising from complications of diarrhoea, occasioned by lack of knowledge. Health education has been shown to help reduce delays in the commencement of home management of diarrhoea. The aim of this study was to assess knowledge of home management of diarrhoea among caregivers of under-fives in Yenagoa Bayelsa State and determine if educational intervention impacted knowledge of diarrhoea management among them.

**Methods:**

a quasi-experimental study which involved an intervention (given health education) and a control (not given health education) group. Using a non-probability convenience sampling technique, 220 participants were recruited. Informed consent was obtained from the participants, after which a 25-item knowledge-assessing structured questionnaire was administered to the participants in both groups to assess their background knowledge of diarrhoea and its home management. Thereafter, only the intervention group was trained using a training guide. At the second contact (one month later), the knowledge of participants of both groups was re-assessed with the 25-item knowledge-assessing structured questionnaire. Responses were scored and then converted to percentages, participants with 70% and above, 50 - 69%, and below 50% were considered to have ´good´, ´fair´ and ´poor´ level of knowledge; this was compared pre- and post-intervention.

**Results:**

at first contact, the knowledge of home management of diarrhoea among the participants was poor in both groups (intervention 9.1%, control 8.2%). However, there was a significant improvement in the knowledge of home management of diarrhoea among those in the intervention group compared to the control group (intervention 95.5%, control 7.3%) (p=0.001), at second contact.

**Conclusion:**

the study shows that health education interventions are effective in strengthening diarrhoea literacy among caregivers of children less than five years of age. Public enlightenment through regular health education of caregivers and the use of mass media is recommended.

## Introduction

Diarrhoea remains a serious public health concern globally and is responsible each year for about 1.3 million childhood deaths worldwide [1,2]. It is widely recognized to be the second leading cause of childhood morbidity and mortality in many developing countries, including Nigeria [1]. Children less than 5 years of age in sub-Saharan Africa experience a median of five episodes of diarrhoea per year, with the highest incidence occurring in those aged 6-23 months [3]. In Nigeria, diarrhoea accounts for over 16% of childhood mortality, causing an estimated 150,000 deaths annually [4]. The WHO recommends early and appropriate fluid replacement and continuous feeding as key interventions to prevent death during acute episodes of diarrhoea [3]. Thus, home treatment has become an essential part of the initial management of diarrhoea in Nigeria and beyond [4]. Despite this, less than 35% of children with diarrhoea receive appropriate home treatment with Oral Rehydration Salts (ORS) and other remedies [5].

This finding has been linked to the low level of knowledge of this cost-effective intervention by mothers and caregivers [4,5]. Various studies have shown that educational interventions have produced significant improvement in mother/caregiver´s knowledge and practice with respect to home care for children with diarrhoeal disease [4,5]. It is therefore imperative to improve caregivers' knowledge of home management strategies of diarrhoea using the veritable tool of health education. Saving the lives of millions of children at risk of death from diarrhoea is possible with a comprehensive strategy that ensures that all children in need receive prompt life-saving measures at home before presentation at the hospital. We therefore sought to determine the level of knowledge and effect of health education on the home management of diarrhoea among caregivers of under-fives attending two tertiary health centres in Yenagoa Local Government Area (LGA), Bayelsa State.

## Methods

**Study design and setting:** this was a quasi-experimental study that involved intervention and control groups. Data were collected at the immunization clinics of the two tertiary health facilities in Yenagoa LGA; the Federal Medical Centre, Yenagoa (FMCY) and the Niger Delta University Teaching Hospital (NDUTH) Okolobiri from January to April 2021. Both immunization clinics attend to 20-30 children every day, excluding weekends and holidays.

**Study population:** mothers/caregivers with under five years old children who presented at the immunization clinics during the study period were recruited after giving consent.

**Inclusion/exclusion criteria:** caregivers with at least one child aged less than 5 years attending the immunization clinic of the selected public health care facilities in Yenagoa LGA. Caregivers who were not available for follow-up were excluded.

**Sample size determination:** the standard formula for interventional studies was used to calculate the minimum sample size [6], with an expected increase of 10% in the level of good knowledge; the expected new proportion would be 18.6%. Setting a significance level of 95% and a power of 80% (0.8) for the study, the sample size was calculated using the formula shown below [6].


$$n={\left[\frac{Z_{\left(\alpha \right)}\sqrt{P_{0}\left(1-P_{0}\right)}-Z_{\left(1-\beta \right)}\sqrt{P_{1}\left(1-P_{1}\right)}}{P_{1}-P_{0}}\right]}^{2}$$

Where n was the minimum sample size required for the study; *P_0_* was the initial proportion of people with a good level of knowledge of home management of diarrhoea (8.6% [4] 0.086); *P_1_* was the new proportion expected as a result of the intervention where a 10% increase in the proportion of mothers/caregivers with a good level of knowledge on home management of diarrhoea is proposed; bringing the new expected proportion (*P_1_*) to 18.6% (0.186) after the intervention. Z(_α_) was the statistic that defines the level of confidence desired in the study (1.96 for significance of 95% CI); Z(_β_) was the statistic that defines the power of the study (- 0.84 for a power of 80%, one-sided). The minimum sample size was approximately 77 participants per group. Considering 40% attrition (resulting from loss to follow-up), 40% of 77 was approximately 31, bringing the minimum sample size to 108 participants per group (77 + 31). The minimum sample size for the study was therefore 216 participants (108 participants in the intervention group and 108 participants in the control group). However, a total of 277 participants were selected for this study and 220 completed the study.

**Data collection:** the two tertiary health facilities in Bayelsa State Federal Medical Centre, Yenagoa (FMCY) and NDUTH were purposively selected. A form of simple random sampling (balloting) was used to assign the institutions as intervention (NDUTH) or control (FMCY). Caregivers with at least one child aged less than 5 years attending the immunization clinic of the selected tertiary health care facilities (NDUTH or FMCY) in Yenagoa LGA who consented to the study were recruited using a non-probability convenience sampling technique. A semi-structured interviewer-administered questionnaire was used to obtain sociodemographic and relevant clinical data on knowledge of home management of diarrhoea (scored attached) from recruited participants at first contact for all participants. Health education on home treatment of diarrhoea was provided for the participants in the intervention group. All participants were invited for a post-intervention impact assessment one month later. The intervention consisted of health education sessions using the training guide adapted from the United Nations International Children's Emergency Fund (UNICEF)/World Health Organization (WHO) training manual on home management of diarrhoea [7] which provides evidence-based guidance for the assessment and home management of diarrhoea. The health education intervention included 1 hour of interactive, theoretical (delivered using tutorials and PowerPoint presentation) and 1 hour of practical sessions (case discussions and role play exercises). The post-intervention impact assessment was conducted with the same version of the questionnaire used pre-intervention, for all the participants (Intervention group and control group). The impact was measured by assessment of the scores (pre- and post-intervention) of subjects on the knowledge of diarrhoea home management. From the 25 items assessing knowledge in the questionnaire, a total of 25 correct answers were expected, and scores were allocated based on the number of correct answers given by each participant. The scores were converted to percentages and participants with 70% and above 50 - 69%, below 50% were considered to have ´good´, ´fair´, and ´poor´ levels of knowledge.

**Data analysis:** each participant was scored based on their response to the 25 items testing knowledge in the study questionnaire. From the 25 items, a total of 25 correct answers were expected, and scores were allocated based on the number of correct answers given by each participant. The scores were converted to percentages and participants with 70% and above were considered to have a ´good´ level of knowledge; participants with percentages between 50 - 69% were categorized as having a ´fair´ level of knowledge and below 50% were classified as having ´poor´ level of knowledge. Categorical variables were expressed as frequencies and proportions, while numerical variables were summarized using means and standard deviations. The chi-square test was used to test relationships between the dependent variables in the study (level of knowledge) and sociodemographic factors of study participants, while a paired t-test was used to test the difference between participants´ mean scores before and after the training. The independent t-test was used to compare differences in the means of scores of participants in the intervention and control groups. A p-value of less than 0.05 was considered statistically significant. Data were analyzed with Statistical Package for Social Sciences (SPSS), version 26.

**Ethical consideration:** the study was approved by the Research and Ethics Committees of the Niger Delta University Teaching Hospital, Okolobiri (NDUTH/REC/2020/0011) and Federal Medical Center Yenagoa (FMCY/REC/ECC/2020/APR/239). All participants gave informed consent.

## Results

The sociodemographic characteristics of all 220 participants studied (110 in intervention and 110 in control groups) are shown in Table 1. One hundred and ten (110) females were in the intervention group; while 108(99.1%) females and 2(0.9%) males were in the control group. The age of all participants ranged from 16 to 46 years, with a mean of 31.0±5.8 years. The mean age of participants in the intervention group was 30.8 ± 6.0 years and for the control group 32.3 ± 5.0 years. The difference was not statistically significant (p = 0.191). Most participants in the control group (86.4%) were residents in urban areas, while 67.3% of those in the intervention group were residents in semi-urban areas. This difference was statistically significant (p = 0.001). At first contact, 10 (9.1%) participants in the intervention group had good knowledge of home management of diarrhoea, and this improved to 105 (95.5%) at second contact. This was statistically significant (p = 0.001). In the control group, 9(8.2%) of the participants had good knowledge of home management of diarrhoea on first contact, this reduced to 8 (7.3%) on second contact a month later (p = 0.908). This is depicted in Table 2. In the Intervention group in Table 3, the mean percentage score on the knowledge of home management of diarrhoea was 47.1 ± 19.8% at first contact but significantly increased to 90.8 ± 13.1% at second contact (an increase of 43.7% above the pre-intervention score) (p = 0.001). An increase of 3.8% was also noticed in the control group on the second visit over the first visit. The mean difference though minimal was also statistically significant (p=0.004). The total knowledge score of participants in the intervention group increased from 73.9 ± 12.9% at in in first contact to 92.2 ± 5.9% at the second contact. This difference was statistically significant (p= 0.001). The total knowledge score however remained the same in the control group on first (72.4 13.4%) and second contact (72.5 13.0%).

**Table 1:** sociodemographic characteristics of participants in the intervention and control groups of the study

Characteristics	Total N =20(%)	Study groups		Test of significance	p-value
		Intervention N = 110 (%)	Control N = 110 (%)		
**Sex**					
Female	218 (99.1)	110 (100.0)	108 (98.2)	2.02^a^	0.498
Male	2 (0.9)	0 (0.0)	2 (1.8)		
**Age group**					
<20 years	8 (3.6)	5 (4.5)	3 (2.7)	4.99^b^	0.173
20 – 29 years	77 (35.0)	42 (38.2)	35 (31.8)		
30 - 39 years	124 (56.4)	55 (50.0)	69 (62.8)		
≥ 40 years	11 (5.0)	8 (7.3)	3 (2.7)		
**Mean age ± SD in years**	31.0 ± 5.8	30.8 ± 6.2	32.3 ± 5.0	1.06^c^	0.191
**Median age (range)**	31.0 (16-46)	30.0 (16 - 44)	31.0 (17 - 46)	1.17^d^	0.167
**Marital status**					
Married	206 (93.6)	101 (91.8)	105 (95.5)	1.22^b^	0.269
Single/widowed	14 (6.4)	9 (8.2)	5 (4.5)		
**Religion**					
Christian	213 (96.8)	107 (97.3)	106 (96.4)	0.15^a^	1.000
^α^Others	7 (3.2)	3 (2.7)	4 (3.6)		
**Residential area**					
Rural	22 (10.0)	16 (14.5)	6 (5.5)	104.36^b^	0.001*
Sub-urban	83 (37.7)	74 (67.3)	9 (8.1)		
Urban	115 (52.3)	20 (18.2)	95 (86.4)		
**Number of children**					
1 – 2 children	124 (56.4)	65 (59.1)	59 (53.6)	0.679^b^	0.712
3 – 4 children	72 (32.7)	34 (30.9)	38 (34.6)		
≥ 5 children	24 (10.9)	11 (10.0)	13 (11.8)		
**Median (range) of children in the family**	2 (1 - 9)	2 (1 - 8)	2 (1 - 9)	0.84^d^	0.402

a Fisher’s exact test, ^b^Chi-square test, ^c^Student T-test, ^d^Median test, *Statistically significant, α- Islam, African Traditional Religion, Eckankar

**Table 2:** level of knowledge of diarrhoea and its home management at first and second contact in both intervention and control groups

Characteristics	Study groups - frequency (%)		percent change (b) - (a)	Chi-square	p-value
	first contact (a)	second contact (b)			
Intervention group					
Poor level	63 (57.3)	3 (2.7)	-54.6	164.43	0.001*
Fair level	37 (33.6)	2 (1.8)	-31.8		
Good level	10 (9.1)	105 (95.5)	86.4		
Control group					
Poor level	57 (51.8)	55 (50.0)	-1.8	0.19	0.908
Fair level	44 (40.0)	47 (42.7)	2.7		
Good level	9 (8.2)	8 (7.3)	-0.9		

* Statistically significant

**Table 3:** mean percentage scores for knowledge of diarrhoea and its home management in the; intervention and control groups (first and second contact)

Domain	Percentage score (mean ± SD)		Change in % mean score	Paired t-test (p-value)
	First contact	Second contact		
Intervention group				
Definition of diarrhoea	71.0 ± 19.0	76.4 ± 16.2	5.8	2.53 (0.013)*
Causes of diarrhoea	73.7 ± 29.8	94.4 ± 16.2	20.9	6.96 (0.001)*
Danger signs of diarrhoea	71.9 ± 24.1	91.6 ± 13.5	21.9	7.28 (0.00)1*
Transmission	81.7 ± 23.4	98.1 ± 7.0	17.2	7.22 (0.001)*
Complication	76.7 ± 21.3	92.6 ± 13.9	17.8	7.56 (0.001)*
Prevention	92.5 ± 17.1	99.8 ± 1.9	7.5	4.25 (0.001)*
Home management	47.1 ± 19.8	90.8 ± 13.1	43.7	18.05 (0.001)*
Total knowledge score	73.9 ± 12.9	92.2 ± 5.9	18.8	14.78 (0.001)*
Control group				
Definition of diarrhoea	68.9 ± 17.1	66.7 ± 14.4	-2.3	1.62 (0.109)
Causes of diarrhoea	80.2 ± 23.9	76.8 ± 26.7	-3.5	1.64 (0.105)
Danger signs of diarrhoea	59.8 ± 28.8	61.0 ± 27.0	0.9	0.49 (0.629)
Transmission	80.8 ± 25.9	81.9 ± 25.1	1.1	0.41 (0.684)
Complication	70.6 ± 24.4	71.4 ± 23.1	0.6	0.28 (0.779)
Prevention	92.8 ± 17.4	92.6 ± 17.7	-0.2	0.15 (0.885)
Home management	45.6 ± 21.8	49.3 ± 18.5	3.7	2.93 (0.004)*
Total knowledge score	72.4 ± 13.4	72.5 ± 13.0	0.1	0.05 (0.957)

* Statistically significant

## Discussion

This current study demonstrates that health education intervention improves the knowledge of caregivers on diarrhoea care. It also demonstrates that the UNICEF/WHO training manual could be used as a basis to develop new short health education programs in settings with poor resources. It confirms that it is possible and useful to impart the principles of diarrhoea home management to a varied group of caregivers with diverse experiences and levels of education looking after children in low-resource settings. This accentuates the universal aspect of the UNICEF/WHO guide, which can be used to build a common understanding in health education programs about the assessment and home management of diarrhoea. The first significant finding of this study is that knowledge of home management of diarrhoea among caregivers of under-fives in Yenagoa LGA is low. More than half of the participants in both intervention and control groups had poor knowledge of home management of diarrhoea while only very few in both groups had good knowledge. This finding is similar to that reported by Adimora *et al*. [8], and Okoh *et al*. [9], Olusola-Feleye [10] in Enugu, Port Harcourt and Lagos Nigeria, also reported low levels of knowledge of home management of diarrhoea among caregivers of under-fives. Some authors attributed the poor level of knowledge of home management of diarrhoea to the low level of education and poor socioeconomic status of the caregivers [6,11]. This is in contrast to the findings in this study, which observed that despite the fact that the participants in the control group had higher levels of education and social class compared to those in the intervention group; more participants in the intervention group had good knowledge of home management of diarrhoea compared to those in the control group at first contact. This could be because semi-urban dwellers could have more time to sit and listen to the health talk compared to the busy city dwellers. This is further accentuated by the fact that the majority of participants got their knowledge of home management of diarrhoea from health workers. There was also no significant association between the participant´s level of knowledge of diarrhoea and its home management and the age, social class, and area of residence or parity of the participants in both groups at first contact. This finding is similar to that reported by Adimora *et al*. [8], Okoh *et al*. [9] and Olusola-Feleye [10].

Knowledge of home management of diarrhoea was noted to have increased by 86.4% among participants in the intervention group at the second visit. This is much higher than reported by Olusola-Faleye [10] in Lagos, with a 31.4% increase in knowledge after health education. This difference in the rate of increase in level of knowledge of home management of diarrhoea in the Lagos study [10] compared to ours could be attributed to the longer duration between the first and second visit in the Lagos study (3 months) when compared to the present one (1 month). This fact is buttressed by findings from Mangala *et al*. [12] in India, where mothers who participated in an intervention study were followed up for two months and two years. There was a significant improvement in the level of knowledge by the mothers after two months which declined when they were assessed two years later [11]. This shows that it is necessary to reinforce this knowledge at frequent short intervals; because health education, an important component of primary health care, is very effective in bringing about significant improvement in knowledge of caregivers.

This study also showed that mean test scores on knowledge of both groups prior to intervention were low. However, on the second visit, the intervention group had a significantly higher mean knowledge score of 90.8 ± 13.1% compared to the control group 3.8%. This significant increase in knowledge of home management of diarrhoea is a result of the focused intervention. This clearly shows that health education significantly improved the knowledge of home management of diarrhoea disease among the participants in the intervention group. This is similar to observations by Olusola-Faleye [10] in Lagos, who reported a significant increase in the mean knowledge score of participants on home management of diarrhoea following intervention. Pahwa *et al*. [13] in India also showed that there was significant improvement in the knowledge, attitude and practice of home management of diarrhoea among the intervention group compared to the control group. Health education is indeed key in improving the knowledge of caregivers on home management of diarrhoea. In the present study, there was a slight but statistically significant improvement (3.8%) in the mean knowledge scores on home management of diarrhoea by participants in the control group at the second visit. This finding is in contrast to what was reported by Olusola-Faleye [10] in Lagos, who noted a significant reduction (-5.4%) in the mean knowledge score of participants in the control group. The reason for this disparity is unclear, but the small improvement in knowledge in the control group could be attributed to the exposure to the subject through completion of the questionnaire which may have prompted the participants to do minimal research, considering that they were city dwellers with high levels of education.

This study shows that if health education programs are properly incorporated, they will improve caregivers´ knowledge of home management of diarrhoea in children, thereby ensuring effective home management practices for diarrhoea, and ultimately reducing under-five mortality. Post-intervention, caregivers felt more self-assured to engage in home management of diarrhoea. Caregivers´ involvement should ideally be strengthened for their children and their own welfare. This intervention can be executed at a low cost and often needs negligible resources, training and skills on the part of the health workers, so should be carried out regularly. Public enlightenment through regular health education of caregivers and the use of mass media is recommended.

**Limitation:** the descriptive cross-sectional nature of this study limits its ability to determine causative factors and can only describe associated factors. A multicenter, multi-regional study may have involved children who may provide a different data set for analysis.

## Conclusion

Our study shows that health education interventions are effective in strengthening diarrhoea literacy among caregivers.

### What is known about this topic

- *Low knowledge level of appropriate use of oral rehydration therapy by caregivers has been attributed to delay in commencing home management of diarrhoea*.

### What this study adds

- *The first significant finding of this study is that knowledge of home management of diarrhoea among caregivers of under-fives in Yenagoa LGA is low and demonstrates that health education intervention improves knowledge of caregivers on diarrhoea care*.
